# Preliminary functional characterization of the LMNA c.929 A > G (p.Gln310Arg) variant identified in a patient with dilated cardiomyopathy

**DOI:** 10.1186/s41065-026-00722-6

**Published:** 2026-08-01

**Authors:** Zikang Han, Yonghao Sheng, Han Sun, Mengchen Sun, Haibin Dong, Lei Gong, Chunxiao Wang, Guangqiang Wang, Lin Zhong

**Affiliations:** 1 Qingdao Medical College, Qingdao university, Qingdao, Shandong China; 2 Department of Cardiology, Yantai Yuhuangding Hospital, Yantai, Shandong China

**Keywords:** Dilated cardiomyopathy, Variant of uncertain significance, *LMNA*, Lamin A/C, ERK1/2

## Abstract

**Background:**

Variants in *LMNA* are established causes of inherited dilated cardiomyopathy (DCM); however, the clinical significance of many rare missense variants remains uncertain. We identified the rare *LMNA* c.929 A > G (p.Gln310Arg) variant in a patient with progressive DCM and performed preliminary variant-specific functional characterization in an AC16 transient overexpression model.

**Methods:**

Duo whole-exome sequencing and copy-number variation analysis were performed in the proband and his son. Wild-type and p.Gln310Arg lamin A expression constructs were transiently transfected into AC16 cells. Flag-tagged protein expression, Flag-based transfection efficiency, and lamin A/C distribution were assessed by western blotting and immunofluorescence staining. Mitochondrial membrane potential and intracellular ROS-associated fluorescence were assessed using JC-1 and DCFH-DA staining, respectively. ERK1/2, JNK, and p38 MAPK phosphorylation was evaluated by western blotting. Hypertrophy-associated *NPPA* and *NPPB* expression was measured by RT-qPCR, and cell area was quantified after phalloidin staining.

**Results:**

The *LMNA* c.929 A > G variant was classified as a variant of uncertain significance. Wild-type and p.Gln310Arg lamin A constructs showed comparable Flag-tagged protein expression and Flag-based transfection efficiency. Expression of p.Gln310Arg lamin A was associated with an increased proportion of cells displaying ring-like lamin A/C staining patterns. Variant-expressing cells showed increased intracellular DCF fluorescence, reduced JC-1 red-to-green fluorescence ratio, and selectively increased ERK1/2 phosphorylation, whereas no significant changes were detected in JNK or p38 MAPK phosphorylation. The same cells also showed a hypertrophy-like phenotype characterized by increased *NPPA* and *NPPB* expression and enlarged cell area.

**Conclusion:**

In an AC16 transient overexpression model, expression of p.Gln310Arg lamin A was associated with altered lamin A/C distribution, increased intracellular ROS-associated fluorescence, reduced mitochondrial membrane potential, selectively increased ERK1/2 phosphorylation, and a hypertrophy-like cellular phenotype. These findings provide preliminary variant-specific functional evidence but do not establish the pathogenicity of the variant or the causal relationships among the observed cellular abnormalities. Further validation in endogenous and physiologically relevant cardac models is required.

**Supplementary Information:**

The online version contains supplementary material available at https://doi.org/10.1186/s41065-026-00722-6.

## Introduction

Dilated cardiomyopathy (DCM) is characterized by left ventricular or biventricular dilation and systolic dysfunction that cannot be explained solely by abnormal loading conditions or coronary artery disease [1, 2]. DCM is a clinically and etiologically heterogeneous disorder in which genetic predisposition, environmental exposures, and acquired myocardial injury may interact to produce a shared phenotype [1, 2]. Contemporary genetic testing can establish a molecular diagnosis in a substantial subset of patients with DCM, although the diagnostic yield varies according to cohort selection, phenotypic stringency, ancestry, and testing strategy [3, 4].

Among the established genes associated with DCM, *LMNA* is clinically important because patients with *LMNA*-related cardiomyopathy frequently develop conduction-system disease, atrial or ventricular arrhythmias, progressive heart failure, and an increased need for heart transplantation [4, 5]. Lamins A and C are type V intermediate filament proteins that constitute major components of the nuclear lamina and contribute to nuclear mechanics, chromatin organization, gene regulation, mechanotransduction, and intracellular signaling [6, 7]. However, the well-established association between *LMNA* and cardiomyopathy at the gene level does not establish the pathogenicity of every rare *LMNA* variant. Interpretation of an individual variant requires the integration of population-frequency data, computational predictions, phenotypic and segregation evidence, and appropriately validated functional findings within the ACMG/AMP framework [8].

Experimental studies have linked lamin A/C dysfunction to impaired nuclear mechanics, nuclear-envelope instability, and increased susceptibility to mechanically induced cellular injury [9, 10]. Dysregulated mitogen-activated protein kinase signaling, including activation of extracellular signal-regulated kinase 1/2, has also been demonstrated in experimental models of *LMNA*-related cardiomyopathy [11]. More recently, patient-specific induced pluripotent stem cell-derived cardiomyocyte models have provided evidence linking lamin A/C deficiency to impaired mitochondrial bioenergetics and increased oxidative stress [12]. Nevertheless, the temporal and causal relationships among altered nuclear organization, oxidative stress, mitochondrial abnormalities, MAPK activation, and pathological cardiac remodeling remain incompletely defined, particularly for individual missense variants of uncertain significance.

In the present study, we identified the heterozygous *LMNA* c.929 A > G (p.Gln310Arg) missense variant in a patient with progressive DCM. Because the available genetic and familial evidence was insufficient to establish pathogenicity, we performed a preliminary variant-specific functional characterization in an AC16 transient overexpression model. Wild-type and p.Gln310Arg lamin A constructs were compared with respect to construct expression and transfection efficiency, lamin A/C subcellular distribution, intracellular ROS-associated fluorescence, mitochondrial membrane potential, MAPK phosphorylation, hypertrophy-associated gene expression, and two-dimensional cell area. The purpose of this study was to determine whether p.Gln310Arg expression was associated with reproducible cellular phenotypes that could support further investigation in more physiologically relevant models, rather than to establish definitive pathogenicity or a causal molecular pathway.

## Materials and methods

### Study participant and clinical assessment

This case-based study included a male patient with clinically diagnosed dilated cardiomyopathy who was treated at Yantai Yuhuangding Hospital. Clinical information was retrospectively obtained from the available medical records, including the patient’s medical and family history, physical examination findings, electrocardiographic and echocardiographic data, laboratory results, treatment, and follow-up. The diagnosis of DCM was based on the presence of left ventricular dilation and systolic dysfunction that could not be adequately explained by abnormal loading conditions or significant coronary artery disease on the basis of the available clinical evaluation, according to contemporary diagnostic criteria [1].

### Genetic testing and variant interpretation

Peripheral blood samples were collected from the proband and his son for genetic analysis. Duo whole-exome sequencing and copy-number variation analysis were performed by Fujun Genetics (Fuzhou, China) using a high-throughput sequencing approach. Genomic DNA was fragmented, adapter-ligated, amplified, and enriched by hybrid capture targeting the coding exons and 20-bp flanking regions of 20,099 genes. Sequence reads were aligned to the GRCh37/hg19 human reference genome. For the proband, the mean sequencing depth was 135.33×, and 98.46% of the targeted coding regions were covered at a depth of at least 20×. Single-nucleotide variants and small insertions or deletions were identified and annotated, and copy-number variants involving two or more consecutive exons were also evaluated. Variant nomenclature was based on the *LMNA* reference transcript NM_170707.3 and followed Human Genome Variation Society recommendations. Variants were interpreted according to the American College of Medical Genetics and Genomics/Association for Molecular Pathology framework [8], incorporating population frequency, computational prediction, reported clinical evidence, and available familial data. The candidate *LMNA* c.929 A > G variant was confirmed by Sanger sequencing.

### Sequence conservation and domain analysis

To evaluate the evolutionary conservation of the affected residue, lamin A/C amino acid sequences from human, *Xenopus laevis*, mouse, rat, pig, and chicken were aligned using DNAMAN software. The amino acid corresponding to human Gln310 was examined across the aligned vertebrate sequences. The position of Gln310 within the lamin A/C protein was evaluated in relation to the established domain organization of A-type lamins.

### Ethics statement

The study protocol was approved by the Medical Ethics Committee of Yantai Yuhuangding Hospital (approval No. 2025 − 843). Written informed consent was obtained from all participating individuals.

### Cell culture

AC16 cells were obtained from Shanghai Zhongqiao Xinzhou Biotechnology Co., Ltd. (Shanghai, China). Cells were cultured in Dulbecco’s modified Eagle medium (DMEM; Gibco, USA) supplemented with 10% fetal bovine serum and 1% penicillin–streptomycin solution (both from Gibco). Cells were maintained at 37 °C in a humidified atmosphere containing 5% CO₂.

### Plasmid constructs and transient transfection

Expression plasmids encoding wild-type lamin A or the p.Gln310Arg variant were obtained from Guangzhou Jisai Biotechnology Co., Ltd. (Guangzhou, China). The lamin A coding sequence was based on the *LMNA* reference transcript NM_170707.3 and cloned into the pcDNA3.1-3×Flag-C expression vector. Both the wild-type and variant constructs encoded lamin A with a C-terminal 3×Flag tag, and the variant construct contained the c.929 A > G nucleotide substitution corresponding to the p.Gln310Arg amino acid change. Four experimental groups were included: untransfected AC16 cells (AC16), cells transfected with the empty pcDNA3.1 vector lacking a Flag tag (NC; empty-vector control), cells transfected with the wild-type lamin A construct (LMNA WT), and cells transfected with the p.Gln310Arg lamin A construct (LMNA MUT).

AC16 cells were seeded in 6-well plates and transfected at approximately 70%–80% confluence. For each well, 2.5 µg of plasmid DNA was transfected using 5 µL of P3000 reagent and 5 µL of Lipofectamine 3000 reagent (Thermo Fisher Scientific, USA). Plasmid DNA was combined with P3000 reagent, and the DNA–P3000 mixture and Lipofectamine 3000 were separately diluted in Opti-MEM reduced-serum medium (Thermo Fisher Scientific). The two solutions were then combined and incubated for 20 min at room temperature to allow complex formation. The resulting transfection complexes were added directly to the cells, which were subsequently maintained at 37 °C in an atmosphere containing 5% CO₂. The culture medium was not replaced after transfection, and cells were harvested 48 h later for the indicated downstream assays. Because the NC vector did not contain a Flag tag, Flag-based transfection efficiency was quantified only in the LMNA WT and LMNA MUT groups as the percentage of Flag-positive cells among the total number of DAPI-positive cells. Transfection-efficiency analysis was performed in five independent transfection experiments.

### Western blot analysis

At 48 h after transfection, cells were lysed in radioimmunoprecipitation assay buffer supplemented with phenylmethylsulfonyl fluoride (PMSF; Sparkjade, Jinan, China). Protein concentrations were determined using a bicinchoninic acid protein assay kit (Yeasen Biotechnology, Shanghai, China). Equal amounts of protein (10 µg per lane) were separated by 10%–15% sodium dodecyl sulfate–polyacrylamide gel electrophoresis and transferred to immunoblotting membranes. Membranes were blocked with 5% non-fat dry milk for 1–2 h at room temperature and subsequently incubated overnight at 4 °C with primary antibodies against the DYKDDDDK tag, phospho-ERK1/2, total ERK1/2, phospho-SAPK/JNK, total SAPK/JNK, phospho-p38 MAPK, and total p38 MAPK (all diluted 1:1,000; Cell Signaling Technology, Danvers, MA, USA). Antibodies against β-tubulin or GAPDH were used as loading controls, as indicated for the corresponding blots. After washing, membranes were incubated with a horseradish peroxidase-conjugated anti-rabbit IgG secondary antibody (1:10,000; Affinity Biosciences, China) for 1 h at room temperature. Immunoreactive bands were visualized using an enhanced chemiluminescence substrate (Thermo Fisher Scientific, USA). For each phosphorylated/total protein pair, the same membrane was stripped and re-probed sequentially with antibodies against the corresponding total protein and loading control. Band intensities were quantified using ImageJ software. Phosphorylated ERK1/2, SAPK/JNK, and p38 MAPK signals were normalized to their corresponding total protein signals, whereas total ERK1/2, total SAPK/JNK, total p38 MAPK, and Flag-tagged lamin A expression were normalized to the loading controls shown in the corresponding blots. Flag-expression and MAPK immunoblotting analyses were performed in three independent cell-transfection experiments.

### RNA extraction and reverse transcription quantitative PCR

At 48 h after transfection, total RNA was extracted from AC16 cells using RNA-Easy Reagent (Vazyme Biotech, Nanjing, China) according to the manufacturer’s instructions. RNA concentration and purity were assessed using a NanoDrop 2000 spectrophotometer (Thermo Fisher Scientific, USA). One microgram of total RNA was treated with gDNA Eraser and reverse-transcribed using the PrimeScript RT reagent Kit with gDNA Eraser (Perfect Real Time; RR047A; Takara Bio, Japan). For genomic DNA removal, 1 µg of total RNA was combined with 2 µL of 5× gDNA Eraser Buffer and 1 µL of gDNA Eraser, adjusted to a final volume of 10 µL with RNase-free water, and incubated at 42 °C for 2 min. The reaction mixture was then supplemented with 1 µL of PrimeScript RT Enzyme Mix I, 1 µL of RT Primer Mix, 4 µL of 5× PrimeScript Buffer 2, and 4 µL of RNase-free water to a final volume of 20 µL. Reverse transcription was performed at 37 °C for 15 min, followed by enzyme inactivation at 85 °C for 5 s.

Quantitative PCR was performed using TB Green Premix Taq (2×; Takara Bio) in a final reaction volume of 25 µL. Each reaction contained 12.5 µL of 2× TB Green Premix Taq, 2 µL of cDNA, 0.5 µL each of the forward and reverse primers (10 µM), and 9.5 µL of RNase-free water. The amplification program consisted of an initial denaturation at 95 °C for 30 s, followed by 40 cycles of 95 °C for 5 s and 60 °C for 30 s. A dissociation-curve analysis was subsequently performed using sequential steps of 95 °C for 15 s, 60 °C for 30 s, and 95 °C for 15 s. The expression levels of NPPA and NPPB were normalized to GAPDH. Normalized expression was displayed as 2^−ΔCt^, where ΔCt was defined as Ct_target−Ct_GAPDH. Statistical analyses of RT-qPCR data were performed using ΔCt values rather than the exponentially transformed 2^−ΔCt2^ values. The primer sequences are listed in Table 1. RT-qPCR analyses were performed in five independent transfection experiments, and technical replicate Ct values were averaged within each biological replicate before calculation of ΔCt.

**Table 1:**
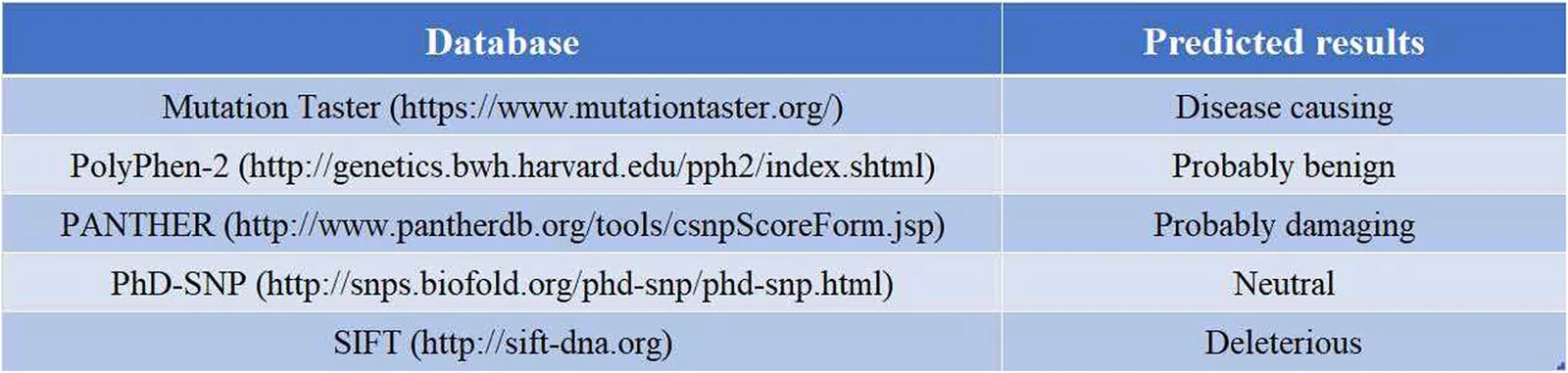
Pathogenicity prediction of LMNA c.929 A > G.

*NPPA* forward: 5′-CGGAAGCTGTTGCAGCCTA-3′

*NPPA* reverse: 5′-GCCCTGGAGCGAGACCGA-3′

*NPPB* forward: 5′-TTTGGGCAGAAGATAGACCG-3′

*NPPB* reverse: 5′-TGGCAGTTGGGCTGAA-3′

*GAPDH* forward: 5′-AGTGCCAGCCTCGTCTCAT-3′

*GAPDH* reverse: 5′-AGGGCCATCCACAGTCTTC-3′

### Phalloidin staining and cell-area quantification

At 48 h after transfection, cells were processed for phalloidin staining using a commercially available staining reagent (Beyotime, Shanghai, China) according to the manufacturer’s instructions. Cells were washed with phosphate-buffered saline (PBS) and fixed with 4% paraformaldehyde for 10 min at room temperature. After fixation, cells were washed with PBS to remove residual paraformaldehyde and incubated with the phalloidin working solution for 1 h at room temperature in the dark to visualize filamentous actin. After staining, cells were washed with PBS, and nuclei were counterstained with DAPI (Beyotime) for 3 min at room temperature. Fluorescence images were acquired using an inverted fluorescence microscope. For each group, three randomly selected, non-overlapping fields were imaged in each independent transfection experiment. Individual cell boundaries were delineated using phalloidin-labeled F-actin, and the two-dimensional cell area was quantified using ImageJ software. Fifty cells were analyzed per group in each independent transfection experiment. The mean cell area calculated from the 50 cells was treated as one biological replicate, and three independent transfection experiments were performed (*n* = 3).

### Immunofluorescence staining and quantification of lamin A/C distribution

At 48 h after transfection, cells were washed with phosphate-buffered saline (PBS) and fixed with 4% paraformaldehyde for 15 min at room temperature. After fixation, cells were permeabilized with PBS containing 0.1% Triton X-100 and 1% bovine serum albumin (BSA) for 1 h at room temperature and subsequently blocked with 10% BSA in PBS for 1 h at room temperature. Cells were incubated overnight at 4 °C with primary antibodies against the DYKDDDDK tag (1:500) and lamin A/C (1:300) (Cell Signaling Technology, Danvers, MA, USA). After washing with PBS, cells were incubated with the appropriate fluorophore-conjugated secondary antibodies for 1 h at room temperature in the dark. Nuclei were counterstained with DAPI (Beyotime, Shanghai, China) for 5 min at room temperature. Fluorescence images were acquired using a fluorescence microscope and analyzed using ImageJ software.

For each experimental condition, three technical replicate wells were analyzed in each independent transfection experiment. Multiple randomly selected, non-overlapping fields were examined in each well until approximately 100 eligible cells had been evaluated. Because the empty pcDNA3.1 vector used in the NC group did not contain a Flag tag, transfection efficiency was evaluated only in the LMNA WT and LMNA MUT groups and was calculated as the percentage of Flag-positive cells among all DAPI-positive cells. A cell was classified as ring-positive when at least one distinct closed or nearly closed annular lamin A/C staining structure with a relatively signal-poor center was observed within or adjacent to the nucleus. In the AC16 and NC groups, all DAPI-positive cells with interpretable lamin A/C staining were included in the analysis, whereas only Flag-positive cells were evaluated in the LMNA WT and LMNA MUT groups. For each condition, the proportion of ring-positive cells was calculated separately for each technical well, and the mean of the three wells was treated as one biological replicate. Five independent transfection experiments were performed (*n* = 5).

### JC-1 staining and assessment of mitochondrial membrane potential

At 48 h after transfection, mitochondrial membrane potential was assessed using a JC-1 assay kit (Solarbio Science & Technology, Beijing, China) according to the manufacturer’s instructions. Cells were washed with phosphate-buffered saline (PBS) and incubated with the JC-1 working solution for 20 min. After staining, cells were washed twice with PBS to remove excess dye. Fluorescence images were acquired using a fluorescence microscope, and the red fluorescence of JC-1 aggregates and the green fluorescence of JC-1 monomers were quantified using ImageJ software. Mitochondrial membrane potential was expressed as the JC-1 red-to-green fluorescence ratio. For each group, fluorescence intensities were measured in randomly selected, non-overlapping fields under identical imaging settings, and the mean red-to-green fluorescence ratio from each independent transfection experiment was treated as one biological replicate. Three independent transfection experiments were performed (*n* = 3).

### DCFH-DA assay for intracellular ROS-associated fluorescence

At 48 h after transfection, intracellular ROS-associated fluorescence was assessed using the cell-permeable probe 2′,7′-dichlorodihydrofluorescein diacetate (DCFH-DA). The culture medium was replaced with fresh medium containing 10 µM DCFH-DA, and cells were incubated for 20 min at 37 °C in the dark. After incubation, cells were washed three times with phosphate-buffered saline (PBS) to remove residual extracellular probe. Cells were subsequently maintained in PBS and immediately imaged using a fluorescence microscope. Intracellular DCF fluorescence intensity was quantified using ImageJ software and used as an indicator of intracellular ROS-associated oxidative activity. Three independent transfection experiments were performed (*n* = 3), and the mean fluorescence value obtained from each independent experiment was treated as one biological replicate. Fluorescence-based assessment of intracellular oxidative status has also been incorporated into recent studies evaluating ROS-scavenging and ROS-responsive biomaterial interventions [13, 14].

### Statistical analysis

RT-qPCR analyses of *NPPA* and *NPPB*, Flag-based transfection efficiency, and quantification of ring-like lamin A/C structures were performed in five independent transfection experiments. Western blotting, DCFH-DA staining, JC-1 staining, and cell-area analyses were performed in three independent transfection experiments. Technical replicates, including replicate wells, microscopic fields, qPCR technical replicates, or individual cells, were averaged within each independent experiment before statistical analysis. Statistical analyses were performed using GraphPad Prism version 9.5 (GraphPad Software, San Diego, CA, USA). Data are presented as the mean ± standard deviation (SD), with individual biological replicate values shown where applicable.

Comparisons between two conditions measured within the same set of independent experiments were performed using a two-tailed paired Student’s *t*-test. For comparisons among three or more conditions generated and analyzed in parallel within the same independent transfection experiments, one-way repeated-measures analysis of variance was used, followed by Tukey’s multiple-comparisons test. Specifically, Flag-tagged lamin A expression and Flag-based transfection efficiency were compared between the LMNA WT and LMNA MUT groups using a two-tailed paired *t*-test, whereas four-group comparisons of molecular and cellular outcomes were analyzed using one-way repeated-measures analysis of variance followed by Tukey’s test. Because several assays included only three independent experiments, formal normality tests were considered insufficiently informative; the distribution of individual values and model residuals was inspected before parametric analysis. All statistical tests were two-sided, and *P* < 0.05 was considered statistically significant. Exact P values are reported in the Results or supplementary statistical table; values below 0.0001 are reported as *P* < 0.0001.

## Results

### Clinical presentation and disease course of the proband

**Fig. 1:**
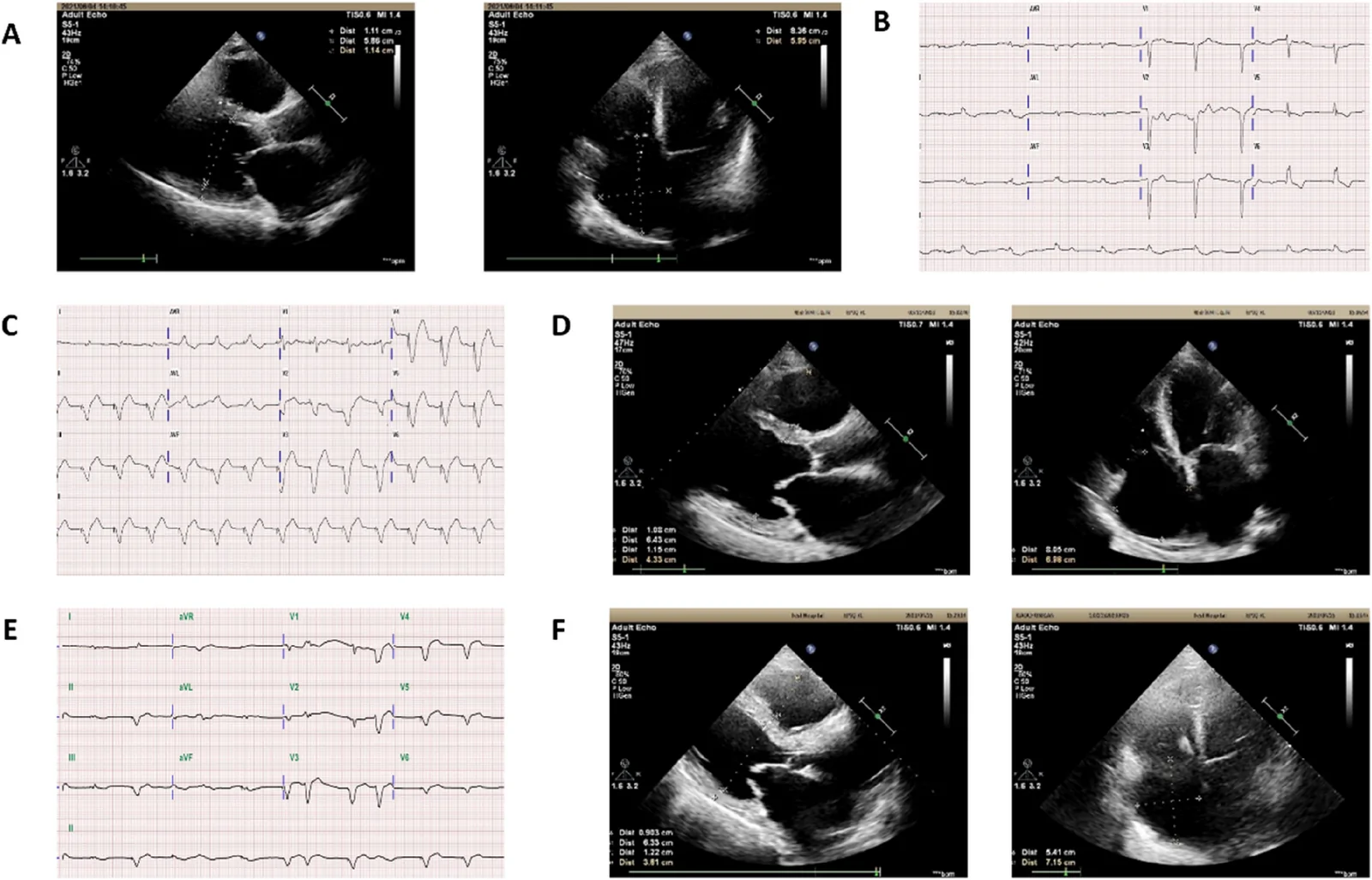
Clinical course of the proband with progressive dilated cardiomyopathy. **A** Echocardiographic findings at the initial clinical evaluation, showing cardiac chamber enlargement and reduced left ventricular systolic function. **B** Twelve-lead electrocardiogram at the initial evaluation. **C** Twelve-lead electrocardiogram after implantation of a single-chamber VVI pacemaker, showing ventricular pacing. **D** Echocardiographic findings at the time of pacemaker implantation. **E** Follow-up electrocardiogram approximately 2 months after pacemaker implantation, showing atrial fibrillation with ventricular pacing. **F** Follow-up echocardiography showing progressive cardiac chamber dilation and reduced left ventricular ejection fraction. LVEF, left ventricular ejection fraction

The proband was a 59-year-old man who was admitted to Yantai Yuhuangding Hospital on June 4, 2021, with a 5-year history of intermittent chest tightness and dyspnea. Physical examination revealed an irregular cardiac rhythm, variable intensity of the first heart sound, a pulse deficit, a murmur over the tricuspid area, and bilateral lower-extremity edema. Baseline echocardiography showed left ventricular systolic dysfunction, with a left ventricular ejection fraction (LVEF) of 40%, together with chamber enlargement: left atrial diameter, 51 mm; left ventricular end-diastolic dimension, 58 mm; right ventricular diameter, 32 mm; and right atrial dimensions, 83 × 59 mm. The left ventricular posterior wall thickness at end-diastole was 11 mm, and severe tricuspid regurgitation was observed (Fig. 1A). The electrocardiogram demonstrated atrial fibrillation, an accelerated junctional rhythm, and complete left bundle branch block (Fig. 1B). Cardiac biomarker measurements showed a high-sensitivity cardiac troponin I level of 68.80 pg/mL, a creatine kinase-MB level of 1.9 ng/mL, and a myoglobin level of 35.5 µg/L. According to the available prior medical records, coronary computed tomography angiography performed at an outside hospital showed no significant coronary artery abnormality.

The patient received sacubitril/valsartan, metoprolol tartrate, spironolactone, furosemide, and rivaroxaban. Despite medical therapy, he continued to experience recurrent chest tightness, dyspnea, and clinically significant arrhythmias. Because of the persistent rhythm and conduction abnormalities, a single-chamber permanent pacemaker programmed in VVI mode was implanted on January 18, 2023; the post-procedural electrocardiogram showed ventricular pacing (Fig. 1C). Echocardiography performed at the time of pacemaker implantation showed a left atrial diameter of 54.4 mm, a left ventricular end-diastolic dimension of 64.3 mm, a right ventricular diameter of 43.3 mm, right atrial dimensions of 80.5 × 69.8 mm, and an LVEF of 43% (Fig. 1D). Two months after pacemaker implantation, the patient again presented with chest tightness and palpitations. Follow-up electrocardiography demonstrated atrial fibrillation with ventricular pacing (Fig. 1E). Follow-up echocardiography showed a left atrial diameter of 60 mm, a left ventricular end-diastolic dimension of 67 mm, a right ventricular diameter of 40 mm, right atrial dimensions of 76 × 56 mm, and a reduction in LVEF to 35% (Fig. 1F). The patient subsequently progressed to end-stage heart failure and died on April 26, 2023.

### Genetic findings and variant interpretation

**Fig. 2:**
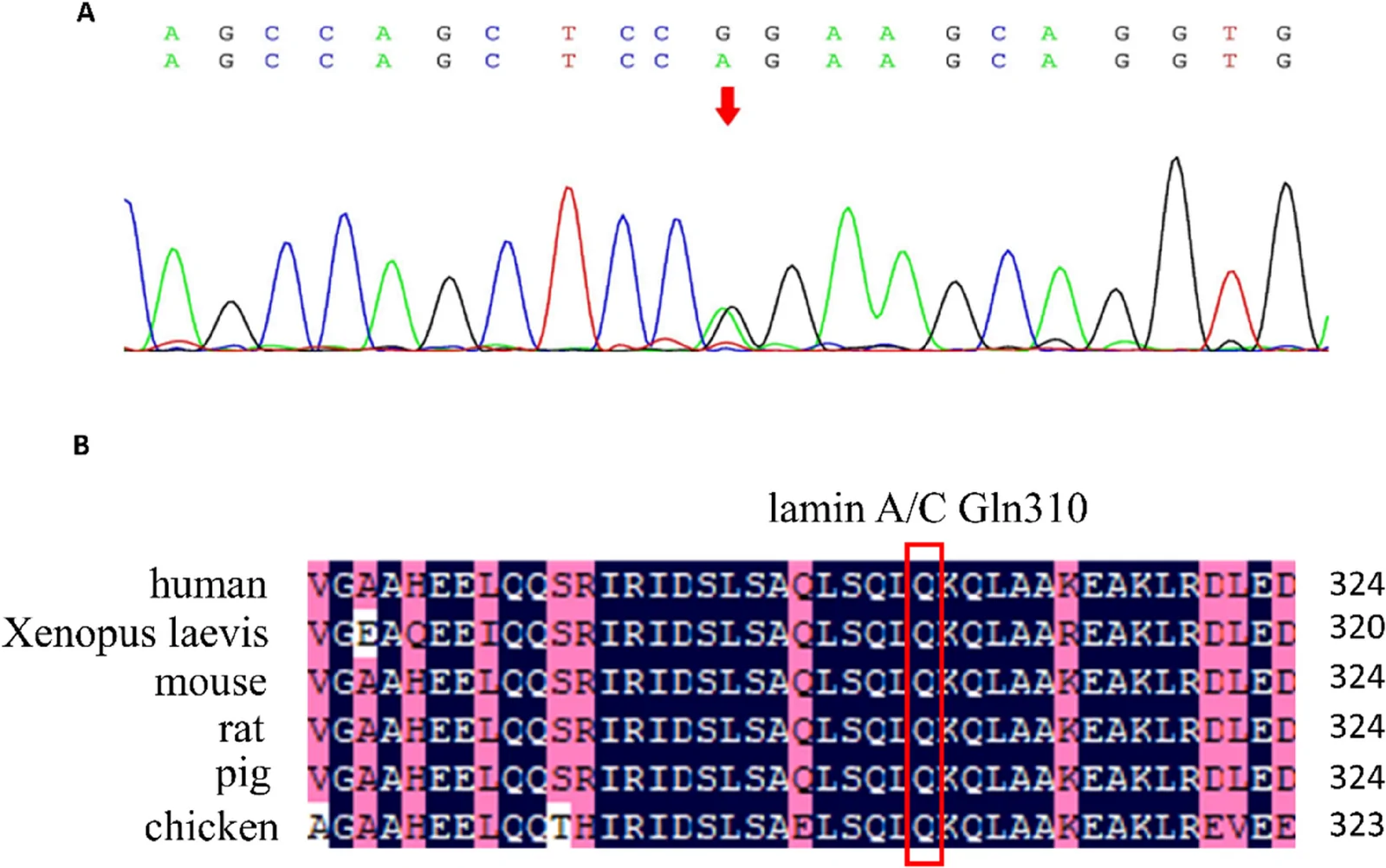
Genetic identification and cross-species conservation of the *LMNA* c.929 A > G (p.Gln310Arg) variant. **A** Sanger sequencing chromatogram confirming the heterozygous *LMNA* c.929 A > G substitution in the proband. The arrow indicates the variant site. **B** Multiple-sequence alignment of lamin A/C protein sequences from human, *Xenopus laevis*, mouse, rat, pig, and chicken. The residue corresponding to human Gln310 is conserved across all six species and is indicated by the box or arrow

Duo whole-exome sequencing identified a heterozygous missense variant in exon 5 of *LMNA*: NM_170707.3:c.929 A > G, predicting the amino acid substitution p.Gln310Arg. Sanger sequencing confirmed the heterozygous *LMNA* c.929 A > G variant in the proband (Fig. 2A), whereas the variant was not detected in his son. The proband’s parents were deceased and unavailable for genetic testing, and no DNA sample was available from his symptomatic brother; consequently, the parental origin of the variant and its cosegregation with disease could not be determined. Family history was notable for a reported diagnosis of DCM in the proband’s mother and reported cardiac enlargement with arrhythmia in one brother; however, these relatives were not genetically tested.

The c.929 A > G substitution is predicted to replace glutamine with arginine at residue 310 of lamin A/C. Domain mapping placed Gln310 within coil 2B of the central α-helical rod domain of lamin A/C. Multiple-sequence alignment showed that the glutamine residue corresponding to human Gln310 was conserved in all six vertebrate species examined, including human, *Xenopus laevis*, mouse, rat, pig, and chicken (Fig. 2B). This conservation supports the potential functional relevance of the residue but does not independently establish the pathogenicity of the p.Gln310Arg substitution. At the time of clinical analysis, the variant was absent from the East Asian datasets of the 1000 Genomes Project, ExAC, and gnomAD, as well as from the testing laboratory’s internal database. In silico prediction tools yielded discordant results: MutationTaster classified the variant as disease causing, PANTHER as probably damaging, and SIFT as deleterious, whereas PolyPhen-2 classified it as probably benign and PhD-SNP as neutral (Table 1). The clinical genetic report documented a REVEL score of 0.726. According to the ACMG/AMP framework applied in the clinical report, the variant was classified as a variant of uncertain significance on the basis of PM2_Supporting and PP3. Thus, the available genetic evidence alone was insufficient to establish pathogenicity.

### Comparable expression and transfection efficiency of wild-type and p.Gln310Arg lamin A constructs in AC16 cells

**Fig. 3:**
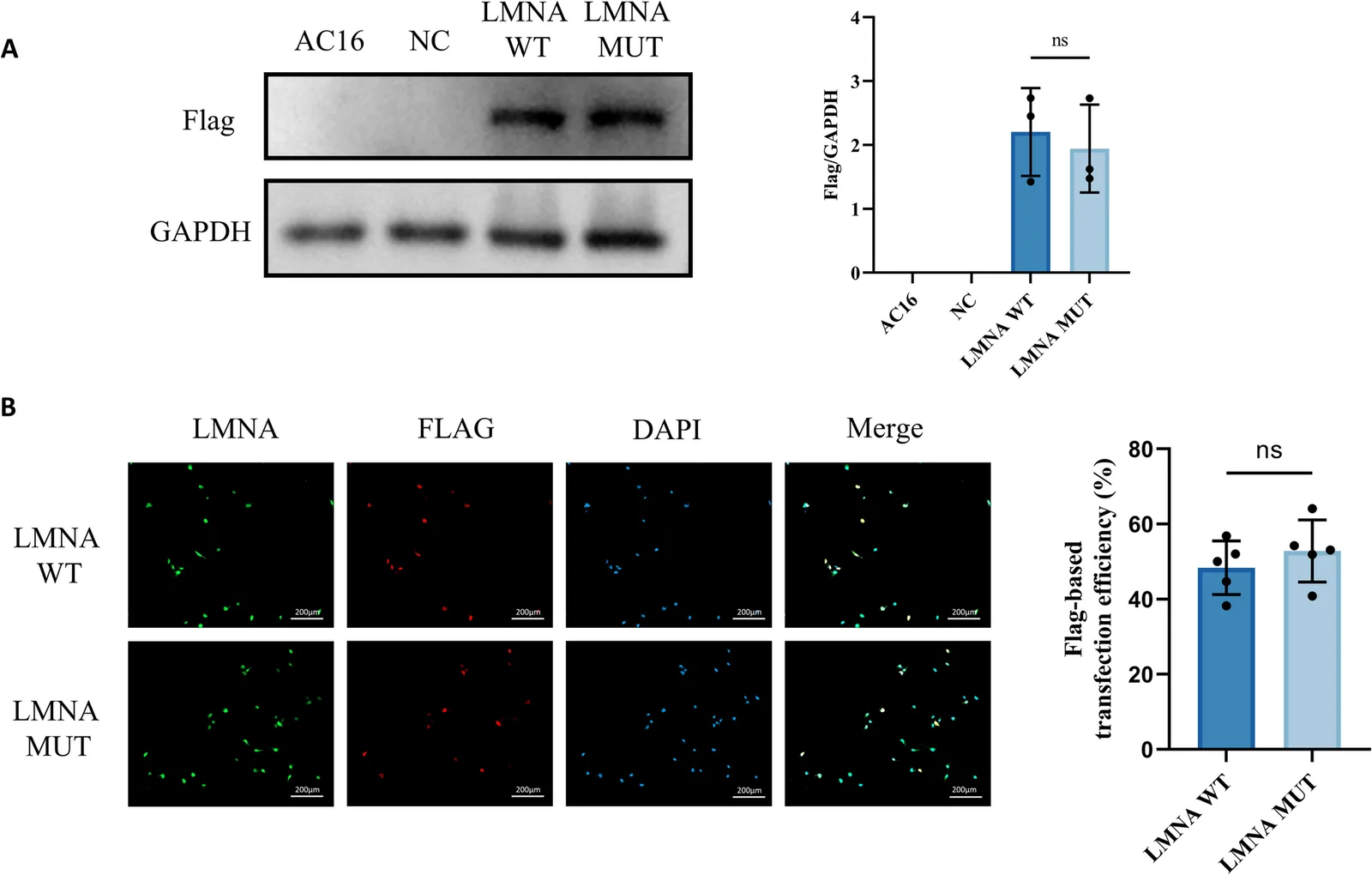
Validation of wild-type and p.Gln310Arg lamin A expression and transfection efficiency in AC16 cells. **A** Representative western blot and densitometric analysis of Flag-tagged lamin A expression in untransfected AC16 cells, cells transfected with the empty pcDNA3.1 vector (NC), cells expressing wild-type lamin A (LMNA WT), and cells expressing p.Gln310Arg lamin A (LMNA MUT). Flag-tagged lamin A expression was normalized to GAPDH. Because the AC16 and NC groups did not encode a Flag-tagged protein, quantitative comparison of Flag-tagged lamin A expression was performed between the LMNA WT and LMNA MUT groups. **B** Representative immunofluorescence images and quantification of Flag-based transfection efficiency in the LMNA WT and LMNA MUT groups. Transfection efficiency was calculated as the percentage of Flag-positive cells among all DAPI-positive cells. Data are presented as the mean ± SD from five independent transfection experiments for transfection-efficiency analysis and from three independent experiments for western blot analysis. Statistical comparisons between LMNA WT and LMNA MUT were performed using a two-tailed paired Student’s *t*-test. ns, not significant

To establish the cellular overexpression model, AC16 cells were left untransfected or transfected with the empty pcDNA3.1 vector, the wild-type lamin A construct, or the p.Gln310Arg lamin A construct. Both the LMNA WT and LMNA MUT constructs encoded lamin A with a C-terminal 3×Flag tag, whereas the empty pcDNA3.1 vector used in the NC group did not contain a Flag tag. Immunoblotting with an anti-Flag antibody detected Flag-tagged lamin A in the LMNA WT and LMNA MUT groups, whereas no Flag signal was detected in the untransfected AC16 or NC groups (Fig. 3A). Densitometric analysis after normalization to GAPDH showed no significant difference in Flag-tagged lamin A expression between the LMNA WT and LMNA MUT groups (two-tailed paired t-test, *P* = 0.6066; Fig. 3A).

Because the NC vector lacked a Flag tag, Flag-based transfection efficiency was evaluated only in the LMNA WT and LMNA MUT groups and was calculated as the percentage of Flag-positive cells among all DAPI-positive cells. The transfection efficiency was 48.34 ± 7.14% in the LMNA WT group and 52.82 ± 8.28% in the LMNA MUT group, with no significant difference between the two groups (two-tailed paired t-test, *P* = 0.4557; Fig. 3B). Together, these results confirmed successful expression of both constructs and supported the comparability of the LMNA WT and LMNA MUT groups for subsequent phenotypic analyses.

### Expression of p.Gln310Arg lamin A was associated with increased ring-like lamin A/C structures

**Fig. 4:**
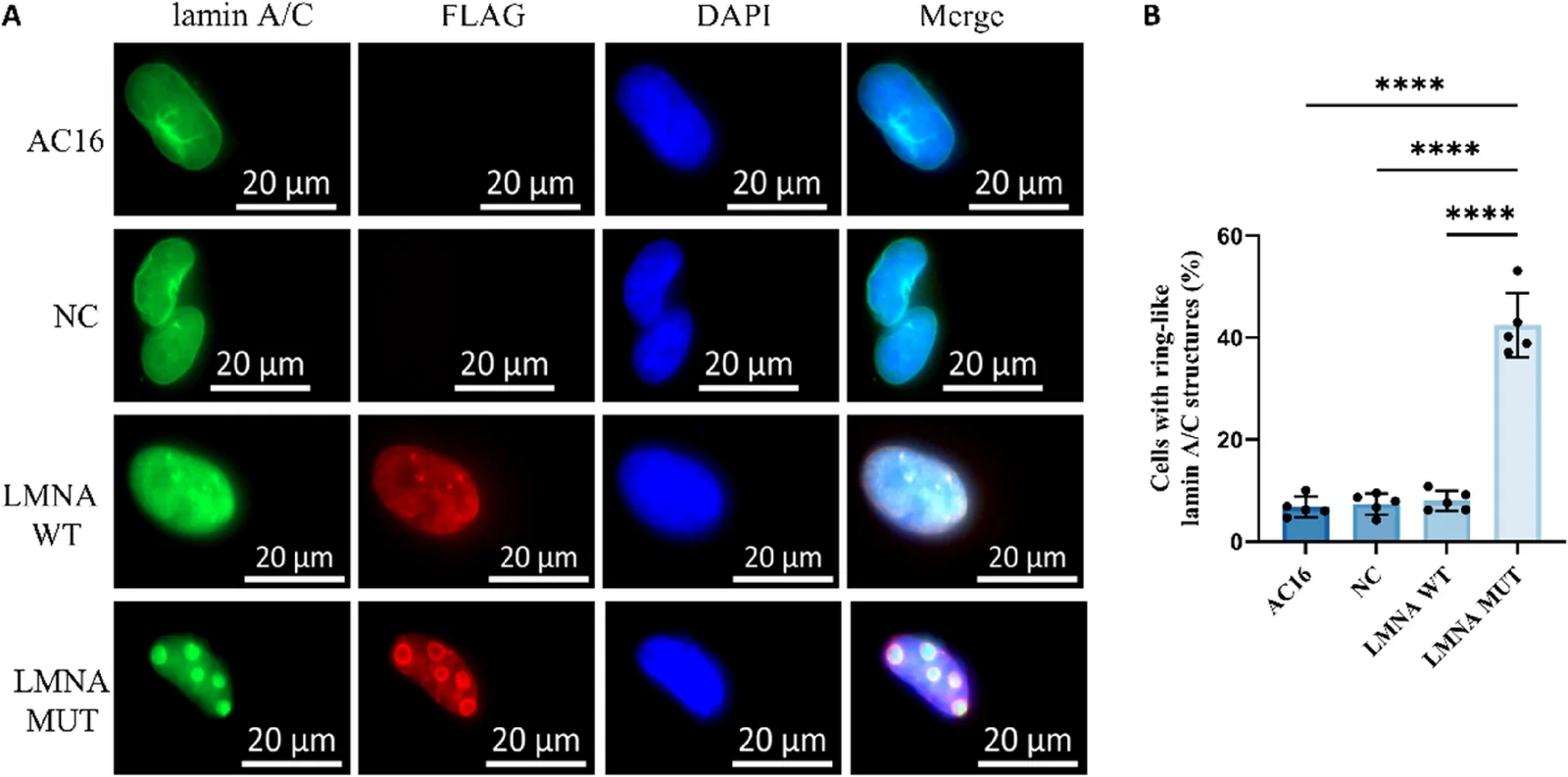
Expression of p.Gln310Arg lamin A was associated with an increased frequency of ring-like lamin A/C structures. **A** Representative immunofluorescence images showing lamin A/C immunoreactivity, Flag-tagged lamin A expression, DAPI-stained nuclei, and merged images in untransfected AC16 cells, cells transfected with the empty pcDNA3.1 vector (NC), cells expressing wild-type lamin A (LMNA WT), and cells expressing p.Gln310Arg lamin A (LMNA MUT). Arrows indicate representative closed or nearly closed ring-like lamin A/C structures. Scale bars, 20 μm. **B** Quantification of cells displaying ring-like lamin A/C staining patterns. In the AC16 and NC groups, DAPI-positive cells with interpretable lamin A/C staining were evaluated; in the LMNA WT and LMNA MUT groups, only Flag-positive cells were included. Each biological replicate represents the mean value from technical replicate wells within one independent transfection experiment. Data are presented as the mean ± SD from five independent transfection experiments. Statistical comparisons were performed using one-way repeated-measures ANOVA followed by Tukey’s multiple-comparisons test. ****P < 0.0001; ns, not significant

To examine whether the p.Gln310Arg variant was associated with altered lamin A/C distribution, lamin A/C and Flag were visualized by immunofluorescence staining in the four experimental groups. In the AC16, NC, and LMNA WT groups, lamin A/C staining was predominantly distributed along the nuclear periphery. In contrast, a subset of Flag-positive cells in the LMNA MUT group displayed distinct closed or nearly closed ring-like lamin A/C structures within or adjacent to the nucleus (Fig. 4A). No overt gross nuclear deformation was apparent on qualitative inspection of the representative images; however, nuclear area and circularity were not quantitatively assessed.

Quantitative analysis showed that ring-positive cells accounted for 6.80 ± 2.01% of cells in the AC16 group, 7.40 ± 2.07% in the NC group, 8.02 ± 1.97% in the LMNA WT group, and 42.46 ± 6.33% in the LMNA MUT group (Fig. 4B). The overall difference among the four groups was significant (one-way repeated-measures ANOVA, F(3,12) = 198.76, *P* < 0.0001). The proportion of ring-positive cells was higher in the LMNA MUT group than in the AC16, NC, and LMNA WT groups (all Tukey-adjusted *P* < 0.0001), whereas no significant differences were observed among the three control groups. These findings indicate that expression of p.Gln310Arg lamin A was associated with an increased frequency of ring-like lamin A/C staining patterns in the AC16 overexpression model.

### Expression of p.Gln310Arg lamin A was associated with increased intracellular ROS-associated fluorescence and reduced mitochondrial membrane potential

**Fig. 5:**
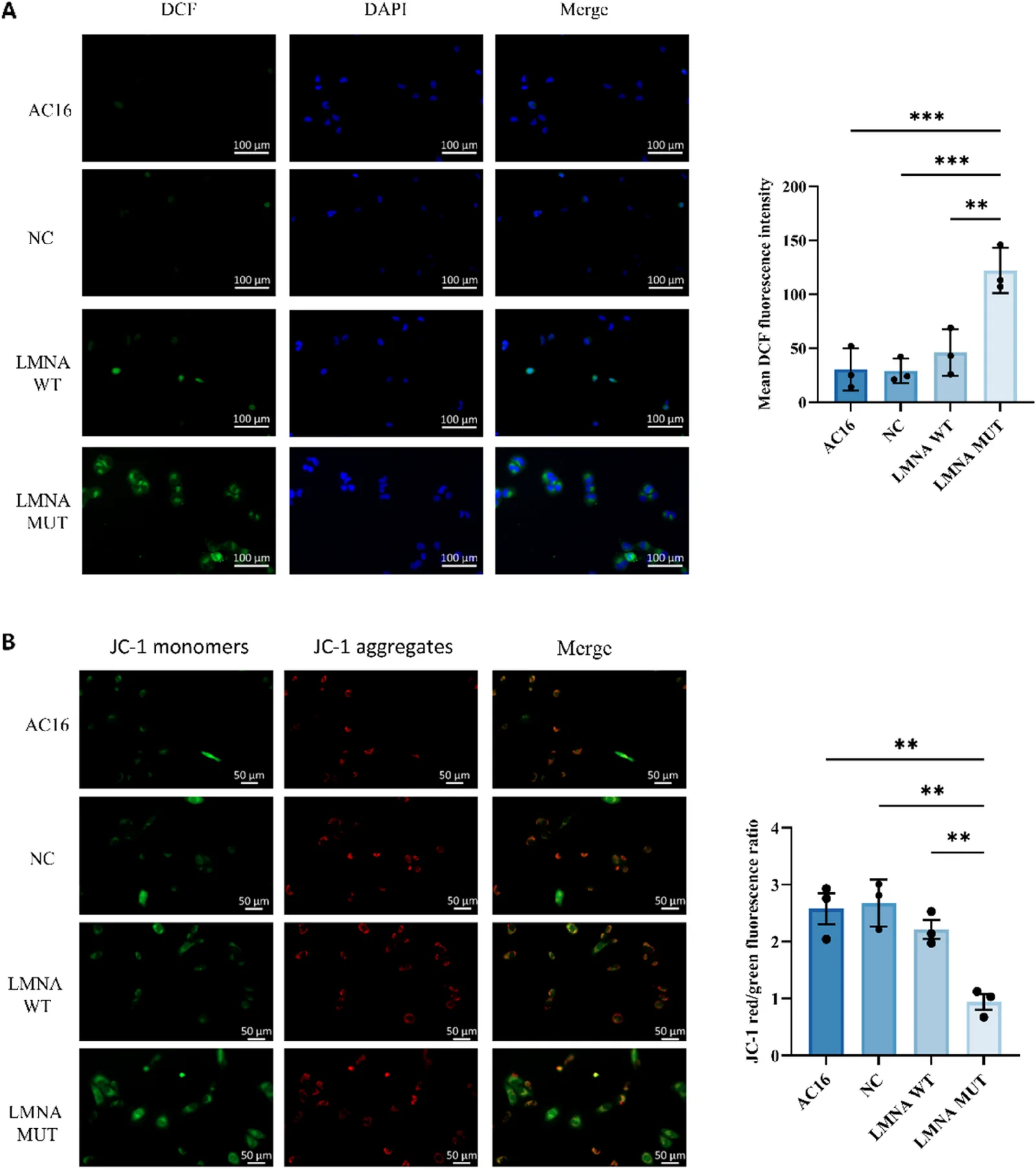
Expression of p.Gln310Arg lamin A was associated with increased intracellular ROS-associated fluorescence and reduced mitochondrial membrane potential. **A** Representative DCFH-DA fluorescence images and quantification of intracellular DCF fluorescence intensity in untransfected AC16 cells, cells transfected with the empty pcDNA3.1 vector (NC), cells expressing wild-type lamin A (LMNA WT), and cells expressing p.Gln310Arg lamin A (LMNA MUT). DCF fluorescence is shown in green, and nuclei were counterstained with DAPI. Scale bars, 100 μm. **B** Representative JC-1 fluorescence images and quantification of the JC-1 red-to-green fluorescence ratio. Green fluorescence represents JC-1 monomers, whereas red fluorescence represents JC-1 aggregates. Scale bars, 50 μm. Data are presented as the mean ± SD from three independent transfection experiments. Statistical comparisons were performed using one-way repeated-measures ANOVA followed by Tukey’s multiple-comparisons test. *P < 0.05 and **P < 0.01

We next assessed intracellular ROS-associated fluorescence and mitochondrial membrane potential in the four experimental groups using DCFH-DA and JC-1 staining, respectively. DCFH-DA staining showed that intracellular DCF fluorescence intensity was significantly higher in the LMNA MUT group than in the untransfected AC16, NC, and LMNA WT groups (Tukey-adjusted *P* = 0.0072, 0.0067, and 0.0176, respectively; Fig. 5A). JC-1 staining demonstrated a lower red-to-green fluorescence ratio in the LMNA MUT group than in the untransfected AC16, NC, and LMNA WT groups (Tukey-adjusted *P* = 0.0081, 0.0060, and 0.0262, respectively; Fig. 5B). The reduced JC-1 red-to-green fluorescence ratio was consistent with mitochondrial membrane depolarization in p.Gln310Arg-expressing cells. Together, these findings indicate that expression of p.Gln310Arg lamin A was associated with increased intracellular oxidative activity and reduced mitochondrial membrane potential in the AC16 overexpression model.

### Expression of p.Gln310Arg lamin A was associated with selectively increased ERK1/2 phosphorylation

**Fig. 6:**
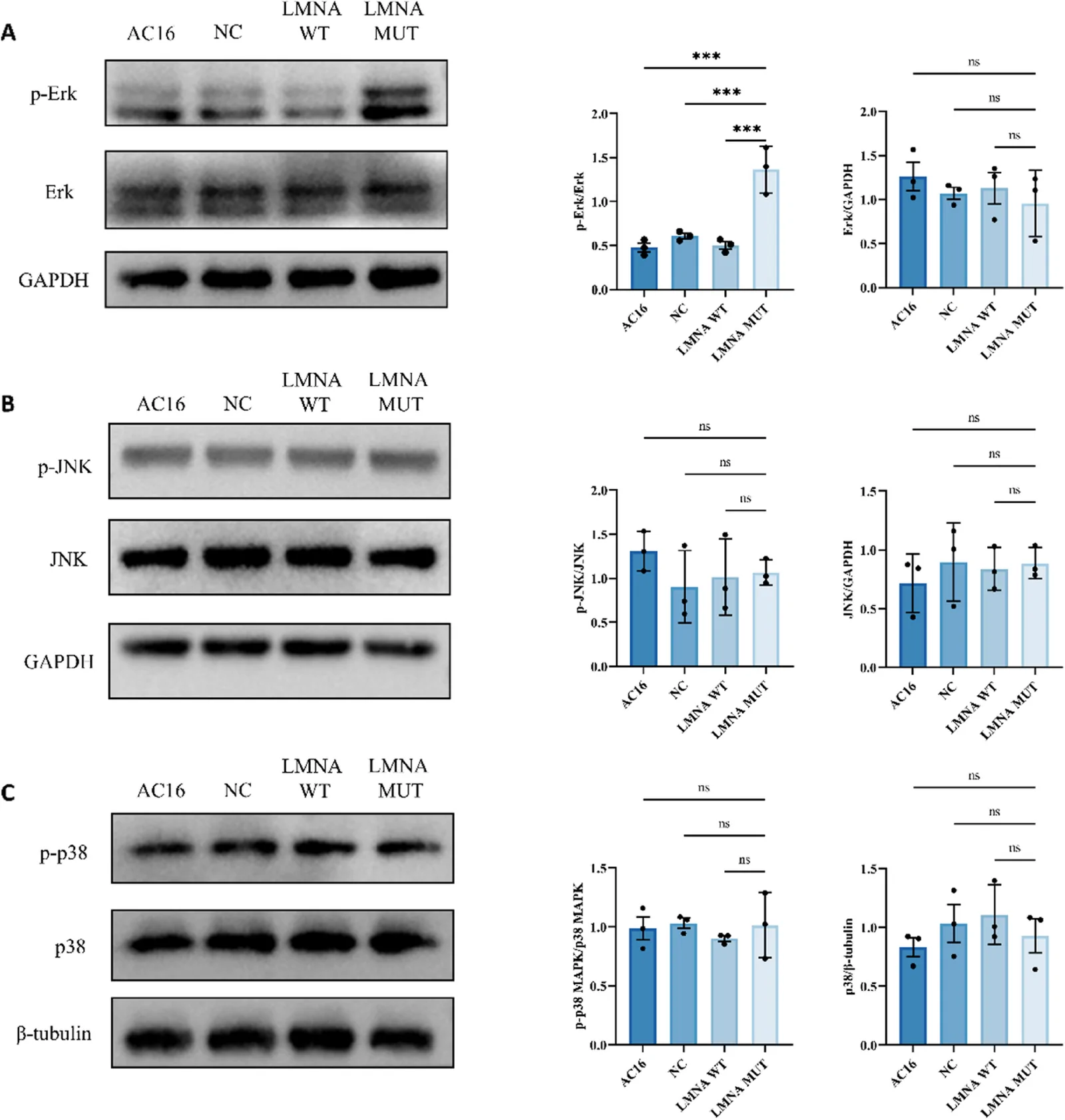
Expression of p.Gln310Arg lamin A was associated with selectively increased ERK1/2 phosphorylation. **A** Representative western blots and densitometric analysis of phosphorylated and total ERK1/2 in untransfected AC16 cells, cells transfected with the empty pcDNA3.1 vector (NC), cells expressing wild-type lamin A (LMNA WT), and cells expressing p.Gln310Arg lamin A (LMNA MUT). Total ERK1/2 was normalized to GAPDH, and phosphorylated ERK1/2 was normalized to total ERK1/2. **B** Representative western blots and densitometric analysis of phosphorylated and total JNK. Total JNK was normalized to GAPDH, and phosphorylated JNK was normalized to total JNK. **C** Representative western blots and densitometric analysis of phosphorylated and total p38 MAPK. Total p38 MAPK was normalized to β-tubulin, and phosphorylated p38 MAPK was normalized to total p38 MAPK. Data are presented as the mean ± SD from three independent transfection experiments. Statistical comparisons were performed using one-way repeated-measures ANOVA followed by Tukey’s multiple-comparisons test. **P < 0.01; ns, not significant

We next examined the phosphorylation status of ERK1/2, JNK, and p38 MAPK in the four experimental groups by western blotting. The phospho-ERK1/2-to-total ERK1/2 ratio was significantly higher in the LMNA MUT group than in the untransfected AC16, NC, and LMNA WT groups (Tukey-adjusted *P* = 0.0015, 0.0035, and 0.0018, respectively; Fig. 6A). Total ERK1/2 abundance normalized to GAPDH did not differ significantly among the four groups.

In contrast, neither the phospho-JNK-to-total JNK ratio nor the phospho-p38 MAPK-to-total p38 MAPK ratio differed significantly among the four groups (Fig. 6B and C). Total JNK normalized to GAPDH and total p38 MAPK normalized to β-tubulin also showed no significant differences among groups. These results indicate that expression of p.Gln310Arg lamin A was associated with selective enhancement of ERK1/2 phosphorylation, without detectable changes in JNK or p38 MAPK phosphorylation under the present experimental conditions. The present data do not establish the temporal or causal relationship between ERK1/2 phosphorylation, oxidative stress, mitochondrial membrane depolarization, and the subsequent hypertrophy-like phenotype.

### Expression of p.Gln310Arg lamin A was associated with a hypertrophy-like phenotype in AC16 cells

**Fig. 7:**
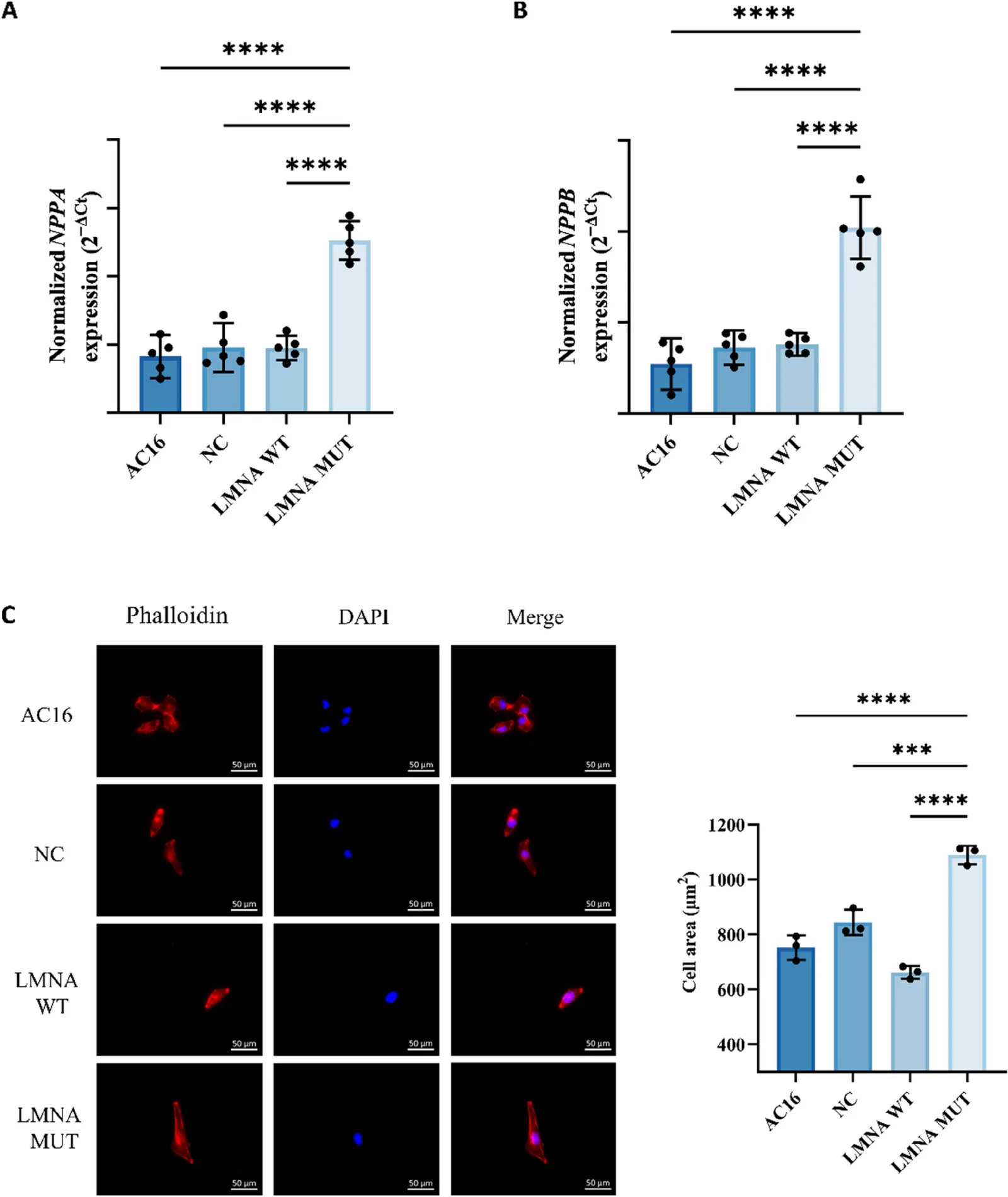
Expression of p.Gln310Arg lamin A was associated with a hypertrophy-like phenotype in AC16 cells. **A–B** Normalized *NPPA* and *NPPB* expression, respectively, in untransfected AC16 cells, cells transfected with the empty pcDNA3.1 vector (NC), cells expressing wild-type lamin A (LMNA WT), and cells expressing p.Gln310Arg lamin A (LMNA MUT), as determined by RT-qPCR. Expression values are displayed as 2 − ΔCt2^{-\Delta Ct}2 − ΔCt, with *GAPDH* used as the reference gene. Statistical analyses were performed using ΔCt values. **C** Representative phalloidin staining images and quantification of two-dimensional cell area. Nuclei were counterstained with DAPI. Scale bars, 50 μm. For RT-qPCR analysis, data are presented as the mean ± SD from five independent transfection experiments. For cell-area quantification, 50 cells were analyzed per group in each independent experiment, and the mean cell area from each experiment was treated as one biological replicate; data are presented as the mean ± SD from three independent transfection experiments. Statistical comparisons were performed using one-way repeated-measures ANOVA followed by Tukey’s multiple-comparisons test. **P < 0.01, ***P < 0.001, and ****P < 0.0001

Finally, we evaluated hypertrophy-associated gene expression and cell area in the four experimental groups. RT-qPCR analysis showed that normalized *NPPA* expression was significantly higher in the LMNA MUT group than in the untransfected AC16, NC, and LMNA WT groups (Tukey-adjusted *P* = 3.23 × 10⁻⁵, 1.32 × 10⁻⁴, and 1.47 × 10⁻⁴, respectively; Fig. 7A). Similarly, normalized *NPPB* expression was significantly increased in the LMNA MUT group compared with the untransfected AC16, NC, and LMNA WT groups (Tukey-adjusted *P* = 4.59 × 10⁻⁵, 7.34 × 10⁻⁴, and 0.0012, respectively; Fig. 7B).

Phalloidin staining further demonstrated that the mean two-dimensional cell area was significantly greater in the LMNA MUT group than in the untransfected AC16, NC, and LMNA WT groups (Tukey-adjusted *P* = 0.0003, 0.0017, and *P* < 0.0001, respectively; Fig. 7C). Cell-area quantification was based on three independent transfection experiments, with 50 cells analyzed per group in each experiment; the mean value from each independent experiment was used for statistical analysis. Together, the increases in *NPPA* and *NPPB* expression and cell area indicate that expression of p.Gln310Arg lamin A was associated with a hypertrophy-like phenotype in the AC16 overexpression model. Although increased ERK1/2 phosphorylation was observed in parallel, the present experiments do not establish that ERK1/2 activation mediated the hypertrophy-like changes.

## Discussion

In this case-based study, we identified the heterozygous *LMNA* c.929 A > G (p.Gln310Arg) missense variant in a patient with progressive DCM characterized by chamber dilation, systolic dysfunction, conduction abnormalities, and atrial arrhythmia. Recent clinical data indicate that LMNA-related cardiomyopathy commonly follows a progressive and arrhythmia-prone course, with substantial morbidity and mortality despite contemporary standard-of-care treatment [15, 16]. Nevertheless, the variant identified in the present study remains classified as a variant of uncertain significance because segregation evidence and definitive functional evidence are insufficient.

Current evidence supports a multifactorial model of LMNA-related cardiomyopathy involving impaired nuclear mechanics, altered chromatin organization, dysregulated intracellular signaling, and metabolic or oxidative stress, although the relative contribution of these processes varies among variants and experimental models [17]. In the present AC16 overexpression model, expression of p.Gln310Arg lamin A was associated with altered lamin A/C distribution, increased intracellular DCF fluorescence, reduced mitochondrial membrane potential, selectively increased ERK1/2 phosphorylation, and a hypertrophy-like cellular phenotype.

The available family history raised the possibility of familial cardiomyopathy, but the segregation evidence was inconclusive. Both parents were deceased and unavailable for genetic testing. The proband’s mother was reported to have had DCM, but no DNA sample or sufficiently detailed clinical records were available for independent verification. The proband’s son, who had no reported cardiac phenotype at the time of evaluation, did not carry the *LMNA* c.929 A > G variant. One of the proband’s brothers reportedly had recurrent chest tightness and dyspnea and had been found to have cardiac enlargement with arrhythmia; however, he did not undergo genetic testing, and no DNA sample was available for analysis. Because parental samples were unavailable and the clinically affected relatives were not genotyped, the parental origin of the variant and its cosegregation with cardiomyopathy could not be determined. The negative result in the unaffected son indicates that the variant was not transmitted to him but does not establish whether the variant was inherited or arose de novo in the proband. Accordingly, the available familial data do not provide sufficient segregation evidence to reclassify the variant, and its inheritance should be described as unknown. Clinical evaluation and appropriately counselled cascade testing of available relatives may help clarify the familial phenotype in future studies.

Recent multicentre data indicate that both variant type and, for selected missense variants, domain location may influence clinical outcomes in *LMNA*-related cardiomyopathy; however, such gene-level genotype–phenotype associations cannot establish the pathogenicity of an individual variant of uncertain significance [18]. Gln310 is located within the coil 2B segment of the central α-helical rod domain of lamin A/C, a region involved in coiled-coil dimerization and higher-order filament assembly [6, 7, 19]. Missense variants within the lamin A/C rod domain have previously been associated with DCM and conduction-system disease, indicating that disruption of this region can be clinically relevant; however, location within the rod domain alone is insufficient to establish the pathogenicity of an individual variant [20]. The p.Gln310Arg substitution replaces a polar uncharged glutamine residue with a positively charged arginine residue. This physicochemical change could potentially affect local coiled-coil interactions, although its structural consequences cannot be determined without direct biophysical or ultrastructural analysis.

Previous studies have shown that selected rod-domain variants can alter lamin organization and nuclear-envelope morphology. For example, Raharjo et al. reported altered lamin assembly and partial emerin mislocalization for DCM-associated lamin A/C variants, whereas Kane et al. observed abnormal nuclear morphology in fibroblasts carrying the p.Asp300Gly rod-domain variant [21–23]. In the present AC16 overexpression model, expression of p.Gln310Arg lamin A was associated with a marked increase in the proportion of cells displaying closed or nearly closed ring-like lamin A/C staining patterns. Ring-positive cells accounted for approximately 41.8% of cells in the LMNA MUT group, compared with approximately 6%–8% in the untransfected, empty-vector, and LMNA WT control groups. These findings support an association between p.Gln310Arg expression and altered lamin A/C subcellular organization. However, the observed staining pattern should not be interpreted as direct evidence of altered protein conformation, defective filament assembly, or nuclear-envelope rupture.

Recent studies using patient-specific *LMNA*-mutant induced pluripotent stem cell-derived cardiomyocytes have provided evidence linking lamin A/C deficiency to mitochondrial bioenergetic impairment and elevated oxidative stress. In an *LMNA* p.Ala388fs model, Qiu et al. observed mitochondrial swelling, reduced maximal and spare respiratory capacity, and increased cellular and mitochondrial ROS levels; activation of SIRT1 partially restored mitochondrial respiration and reduced ROS accumulation [12]. In the present AC16 overexpression model, expression of p.Gln310Arg lamin A was associated with increased intracellular DCF fluorescence and a reduced JC-1 red-to-green fluorescence ratio, consistent with enhanced intracellular oxidative activity and mitochondrial membrane depolarization. These findings are directionally consistent with mitochondrial stress reported in other *LMNA* disease models; however, the present assays do not establish generalized mitochondrial dysfunction or altered energy metabolism. Moreover, DCFH-DA provides a broad redox-sensitive fluorescence signal and does not identify the specific reactive species or their subcellular source; therefore, the increased DCF fluorescence cannot be attributed specifically to mitochondrial ROS. Because ROS-associated fluorescence and mitochondrial membrane potential were measured at the same post-transfection time point, it remains unclear whether oxidative stress preceded mitochondrial depolarization, resulted from it, or developed in parallel. Time-course analyses and intervention experiments using antioxidants, mitochondrial-targeted ROS scavengers, or modulators of mitochondrial function would be required to determine whether these abnormalities contribute causally to ERK1/2 phosphorylation or the hypertrophy-like phenotype.

In the present AC16 overexpression model, expression of p.Gln310Arg lamin A was accompanied by increased *NPPA* and *NPPB* transcript levels and an enlarged two-dimensional cell area, supporting the presence of a hypertrophy-like cellular phenotype. These readouts are associated with cardiac stress and hypertrophic remodeling but do not by themselves establish mature cardiomyocyte hypertrophy or its molecular cause. MAPK dysregulation has been implicated in experimental models of *LMNA*-related cardiomyopathy, although the activation pattern varies among pathway branches and experimental settings. Muchir et al. reported that ERK1/2 and JNK signaling was activated in the hearts and isolated cardiomyocytes of *Lmna*^H222P/H222P^ mice before the development of advanced cardiac dysfunction, suggesting that MAPK abnormalities may occur early in this specific disease model [11]. In the same model, pharmacological inhibition of MEK–ERK signaling reduced ERK phosphorylation, attenuated left ventricular dilation, and improved cardiac function, whereas genetic depletion of *Erk1* provided additional evidence that pathological ERK1/2 activity contributes to disease progression [24, 25].

Consistent with the involvement of ERK signaling in selected laminopathy models, the phospho-ERK1/2-to-total ERK1/2 ratio was increased in p.Gln310Arg-expressing AC16 cells, whereas total ERK1/2 abundance was unchanged. In contrast, no significant changes were detected in the phospho-JNK-to-total JNK or phospho-p38-to-total p38 ratios under the present experimental conditions. This selective pattern differed from that reported in the *Lmna*^H222P/H222P^ model and may reflect differences in the variant, cell type, expression system, disease stage, or timing of analysis. Although ERK1/2 phosphorylation, *NPPA/NPPB* expression, and cell area were increased in parallel, the present data do not demonstrate that ERK1/2 activation mediated the hypertrophy-like phenotype. Establishing such a relationship would require time-course studies and rescue experiments using MEK–ERK inhibition or genetic pathway modulation.

A strength of this study is the integration of detailed clinical and genetic observations with several complementary cellular readouts, including lamin A/C distribution, intracellular redox-sensitive fluorescence, mitochondrial membrane potential, MAPK phosphorylation, hypertrophy-associated gene expression, and cell area. The side-by-side comparison of the WT and MUT constructs, together with comparable Flag expression and transfection efficiency, reduced the likelihood that the observed differences were simply caused by unequal plasmid delivery or gross differences in construct expression. Nevertheless, the present findings should primarily be regarded as preliminary variant-specific functional observations.

Several limitations should be acknowledged. First, the clinical and genetic observations were derived from a single proband, and no additional unrelated carriers were identified. A coronary CTA performed at an outside hospital was reported to show no significant coronary abnormality, making obstructive coronary artery disease less likely; however, detailed source images were unavailable for re-evaluation, and invasive coronary angiography was not performed. Cardiac magnetic resonance imaging and endomyocardial biopsy were also not performed; therefore, myocardial fibrosis patterns and some inflammatory, infiltrative, or other non-genetic causes could not be comprehensively evaluated. In addition, segregation analysis was incomplete because both parents were unavailable and the reportedly affected brother was not genotyped. The inheritance of the variant, its cosegregation with cardiomyopathy, penetrance, and variable expressivity therefore remain unknown.

Second, all functional analyses were performed in AC16 cells using transient plasmid overexpression. Although this model permits preliminary comparison of WT and variant constructs, it does not reproduce endogenous heterozygous expression, native genomic regulation, cardiomyocyte maturation, or the multicellular environment of the human heart. Supraphysiological expression may amplify cellular stress or produce phenotypes that would not occur at endogenous protein levels. Comparable expression and transfection efficiency between the WT and MUT groups improve internal comparability but do not eliminate this limitation. Moreover, the constructs encoded lamin A with a C-terminal 3×Flag tag. Because lamin A undergoes processing at its C terminus, an effect of the tag on protein maturation or localization cannot be excluded, particularly when interpreting the ring-like lamin A/C staining pattern.

Third, the experiments were observational and were performed at a single post-transfection time point. No antioxidant, MEK–ERK inhibitor, or genetic rescue experiment was performed. Accordingly, the temporal and causal relationships among altered lamin A/C distribution, increased intracellular oxidative activity, mitochondrial membrane depolarization, ERK1/2 phosphorylation, and the hypertrophy-like phenotype remain unresolved. DCFH-DA and JC-1 provide limited readouts of cellular redox activity and mitochondrial membrane potential, respectively; mitochondrial respiration, ATP production, mitochondrial ultrastructure, mitochondrial-specific ROS, nuclear-envelope integrity, and mechanical properties were not assessed. Although RT-qPCR analyses, Flag-based transfection-efficiency assessment, and ring-like lamin A/C quantification were performed in five independent transfection experiments, western blotting, DCFH-DA staining, JC-1 staining, and cell-area analyses were based on three independent transfection experiments, which limits statistical precision and the assessment of biological variability for these assays.

Future studies should prioritize validation using untagged, isogenic CRISPR/Cas9 knock-in models and patient-derived or gene-corrected induced pluripotent stem cell-derived cardiomyocytes. Expanded clinical evaluation of available relatives and identification of additional unrelated carriers would be necessary to strengthen genotype–phenotype and segregation evidence. Time-course and rescue experiments using antioxidants and MEK–ERK pathway inhibition, together with measurements of mitochondrial respiration, ATP production, nuclear-envelope integrity, calcium handling, electrophysiology, and contractile function, would help determine whether the observed cellular abnormalities are causally related. Until such evidence becomes available, *LMNA* c.929 A > G (p.Gln310Arg) should remain classified as a variant of uncertain significance.

## Conclusion

The heterozygous *LMNA* c.929 A > G (p.Gln310Arg) variant was identified in a patient with progressive DCM and remains classified as a variant of uncertain significance. In an AC16 transient overexpression model, expression of p.Gln310Arg lamin A was associated with altered lamin A/C distribution, increased intracellular ROS-associated fluorescence, reduced mitochondrial membrane potential, selectively increased ERK1/2 phosphorylation, and a hypertrophy-like cellular phenotype. These findings provide preliminary variant-specific functional evidence but do not establish the pathogenicity of the variant or a causal molecular pathway. Additional segregation data and validation in untagged isogenic knock-in or patient-derived cardiomyocyte models are required before the clinical significance of this variant can be reconsidered.

## Supplementary Information

Supplementary Material 1.

Supplementary Material 2.

Supplementary Material 3.

Supplementary Material 4.

## Data Availability

No datasets were generated or analysed during the current study.

## Abbreviations

DCM: Dilated cardiomyopathy
LMNA: Lamin A/C gene
WT: Wild type
MUT: p.Gln310Arg lamin A construct group
NC: Empty-vector control
ECG: Electrocardiography
LVEF: Left ventricular ejection fraction
LA: Left atrium
LVEDD: Left ventricular end-diastolic dimension
RVd: Right ventricular diameter
LVPWd: Left ventricular posterior wall thickness at end-diastole
WES: Whole-exome sequencing
CNV: Copy-number variation
ACMG/AMP: American College of Medical Genetics and Genomics/Association for Molecular Pathology
VUS: Variant of uncertain significance
RT-qPCR: Reverse transcription quantitative polymerase chain reaction
NPPA: Natriuretic peptide A gene
NPPB: Natriuretic peptide B gene
ANP: Atrial natriuretic peptide
BNP: B-type natriuretic peptide
GAPDH: Glyceraldehyde-3-phosphate dehydrogenase gene
ROS: Reactive oxygen species
DCFH-DA: 2′,7′-dichlorodihydrofluorescein diacetate
DCF: 2′,7′-dichlorofluorescein
MMP: Mitochondrial membrane potential
MAPK: Mitogen-activated protein kinase
ERK1/2: Extracellular signal-regulated kinase 1/2
JNK: c-Jun N-terminal kinase
iPSC-CMs: induced pluripotent stem cell-derived cardiomyocytes
